# Salt-dependent regulation of a CNG channel subfamily in Arabidopsis

**DOI:** 10.1186/1471-2229-9-140

**Published:** 2009-11-27

**Authors:** Annette Kugler, Barbara Köhler, Klaus Palme, Patricia Wolff, Petra Dietrich

**Affiliations:** 1 Molecular Plant Physiology, Department Biology, University of Erlangen, Erlangen, Germany; 2 Institute of Biochemistry and Biology, University of Potsdam, Potsdam-Golm, Germany; 3 Institute of Biology II/Botany, Faculty of Biology, University of Freiburg, Freiburg, Germany

## Abstract

**Background:**

In *Arabidopsis thaliana*, the family of cyclic nucleotide-gated channels (CNGCs) is composed of 20 members. Previous studies indicate that plant CNGCs are involved in the control of growth processes and responses to abiotic and biotic stresses. According to their proposed function as cation entry pathways these channels contribute to cellular cation homeostasis, including calcium and sodium, as well as to stress-related signal transduction. Here, we studied the expression patterns and regulation of *CNGC19 *and *CNGC20*, which constitute one of the five CNGC subfamilies.

**Results:**

GUS, GFP and luciferase reporter assays were used to study the expression of *CNGC19 *and *CNGC20 *genes from *Arabidopsis thaliana *in response to developmental cues and salt stress. *CNGC19 *and *CNGC20 *were differentially expressed in roots and shoots. The *CNGC19 *gene was predominantly active in roots already at early growth stages. Major expression was observed in the phloem. *CNGC20 *showed highest promoter activity in mesophyll cells surrounding the veins. Its expression increased during development and was maximal in mature and senescent leaves. Both genes were upregulated in the shoot in response to elevated NaCl but not mannitol concentrations. While in the root, *CNGC19 *did not respond to changes in the salt concentration, in the shoot it was strongly upregulated in the observed time frame (6-72 hours). Salt-induction of *CNGC20 *was also observed in the shoot, starting already one hour after stress treatment. It occurred with similar kinetics, irrespective of whether NaCl was applied to roots of intact plants or to the petiole of detached leaves. No differences in K and Na contents of the shoots were measured in homozygous T-DNA insertion lines for *CNGC19 *and *CNGC20*, respectively, which developed a growth phenotype in the presence of up to 75 mM NaCl similar to that of the wild type.

**Conclusion:**

Together, the results strongly suggest that both channels are involved in the salinity response of different cell types in the shoot. Upon salinity both genes are upregulated within hours. *CNGC19 *and *CNGC20 *could assist the plant to cope with toxic effects caused by salt stress, probably by contributing to a re-allocation of sodium within the plant.

## Background

Salinity has become a major constraint in crop production. Understanding the mechanisms, which enable growth under saline conditions is of high scientific and agricultural interest [1,2]. Sodium uptake and distribution within the plant is a major determinant for the salt sensitivity of a plant. Sustained exposure to elevated salt concentrations leads to the transfer and accumulation of NaCl in the shoot tissue, where it can inhibit leaf growth. Prevention of Na^+ ^entry into the root, transport to and allocation within the leaf, and sequestration into the vacuole are strategies with which plants cope with high salt environment. Accordingly, the overexpression of the vacuolar Na^+^/H^+ ^antiporter NHX1, for instance, improves salt-tolerance in Arabidopsis [3]. Within the shoot, ion allocation can vary between cell types as found in mesophyll and epidermis of barley and wheat, where differences for K^+ ^and Cl^- ^were measured [4,5]. Na^+ ^can either be retained in older leaves reducing transport to young organs or translocated to petioles and leaf margins to protect the lamina from excessive entry of salt as described for *Medicago citrine *and *Ricinus communis *[6,7]. Hence, control of Na^+ ^and K^+ ^fluxes on the whole plant level guarantees the maintenance of a high cytosolic K^+^/Na^+^-ratio, which is crucial for growth in saline soils. In Arabidopsis, transporters contributing to Na^+ ^homeostasis include plasma membrane (SOS1) and vacuolar Na^+^/H^+ ^antiporters (e.g. NHX1), and the plasma membrane uniporter HKT1 [2].

*AtSOS1 *is expressed in epidermal cells at the root tip and in xylem parenchyma cells of roots and shoots [8]. Altogether, data showed that SOS1 controls Na^+ ^extrusion out of the root and long-distance transport, limiting Na^+ ^accumulation in plant cells. The ability of tomato (*Solanum lycopersicum) *plants to retain Na^+ ^in the stems, and thus to prevent Na^+ ^from reaching the photosynthetic tissues, is largely dependent on the function of *Sl*SOS1, the functional homolog of *At*SOS1 [9].

While NHX1 and SOS1 export Na^+ ^from the cytosol on the expense of the proton gradient, Na^+ ^entry follows its electrochemical gradient. Members of two gene families, the high affinity K^+ ^transporter family HKT, and the cyclic nucleotide-gated ion channel family, CNGC, have been shown to mediate Na^+ ^uptake and regulation of long distance transport. Proteins belonging to the HKT family control Na^+ ^unloading in the xylem of Arabidopsis, rice and wheat [1], and therefore control the long-distance transport of Na^+ ^to the leaf. The Arabidopsis genome contains a single *HKT *homolog, *AtHKT1*, which belongs to the subfamily of HKT transporters that encode low affinity Na^+ ^uniporters. Loss-of-function mutations in *At*HKT1 render plants Na^+ ^hypersensitive and disturb the distribution of Na^+ ^between roots and shoots.

Members of the cyclic nucleotide-gated channel (CNGC) family belong to the group of nonselective cation channels and enable the uptake of Na^+^, K^+^, and Ca^2+^[10]. CNG channels are assumed to activate upon binding of cellular cAMP or cGMP to the ligand-binding site. Within the C-terminus of the channel, a partially overlapping binding domain for calmodulin allows Ca^2+^-calmodulin binding and is proposed to destabilize the open state. Functional expression of plant CNG channels in *Xenopus *oocytes or animal cell lines has not been reproducibly successful; hence a detailed biophysical characterization of these channels including their gating and permeation characteristics still remains to be performed. The Arabidopsis CNGC gene family comprises 20 members [11]. Phenotypical analysis of loss-of-function mutants showed that members play a role in plant growth and the response to pathogen attack [10]. CNGC10 is involved in Arabidopsis' tolerance towards salt. Mature plants of *CNGC10 *antisense lines were more sensitive to salt stress and contained higher Na^+ ^concentrations in shoots compared with wild-type [12]. In contrast, salt-grown seedlings of the antisense lines developed longer roots compared to the wild type. Likewise, *cngc3 *mutant seedlings showed slightly enhanced growth in the presence of elevated NaCl or KCl concentrations compared to wild type plants [13]. So far, members tested have been localized in the plasma membrane [13-16], suggesting a direct function in cation entry into the cell.

In this study, we show that both *CNGC19 *and *CNGC20 *respond to salinity with increased gene activity and accumulation of transcripts in the shoot. Salt treatment of roots or cut leaves induced the shoot regulation of *CNGC20*, suggesting that NaCl itself is the root-to-shoot signal. Although the loss of either channel did not lead to a salt-related growth phenotype, the strong upregulation by NaCl underlines their role in the salinity response, which is discussed on the basis of their distinct expression pattern.

## Results and Discussion

We investigated the expression pattern and regulation by salt stress of the group IVA of Arabidopsis CNG channels [11], consisting of *CNGC19 *and *CNGC20*. Both genes are arranged in tandem on chromosome 3. On the amino acid level, the two proteins share 73% identity.

### Distinct expression patterns of *CNGC19 *and *CNGC20*

**Figure 1:**
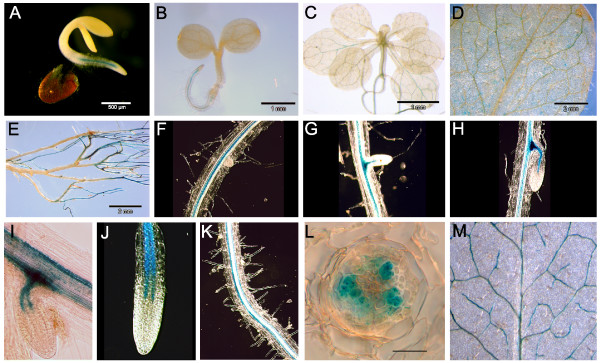
***CNGC19 *expression in vascular tissues**. GUS staining of plants at different ages carrying the *CNGC19p:GUS *construct: 2 (A), 4 (B), 18 (C), 39 (D, E) days after stratification. (F-H) Pictures of developing lateral roots where blue staining is not detected before vasculature formation. (I-K) GUS staining visible in the phloem strands of the root. (L) Cross section of the root with GUS staining in the phloem but not in xylem cells of the stele. The bar represents 20 μm. (M) Part of an adult leaf showing GUS staining in the vasculature. Note, that the plant shown in (M) has been grown on salt-containing agar (see Fig. 3).

The age- and tissue-dependent expression pattern was visualized in plants expressing β-glucuronidase under the control of the CNG channel promoter. In case of *CNGC19*, blue staining was visible starting one day after the radicle's emergence from the seedling. Promoter activity was detected mainly in roots and to a lesser extent in shoots of plants of different developmental stages (Fig. 1A-E). *CNGC19 *showed expression only in the vasculature, and was observed during the development of lateral roots, as soon as the stelar long-distance transport system develops. It was not detected in root meristematic tissue (Fig. 1F-H). The staining of two strands within the stele indicated that the expression is located in phloem tissue (Fig. 1I-K). This was supported by the staining of root cross sections (Fig. 1L). In the shoot, *CNGC19 *promoter activity was observed in the vasculature, too (Fig. 1C, M).

**Figure 2:**
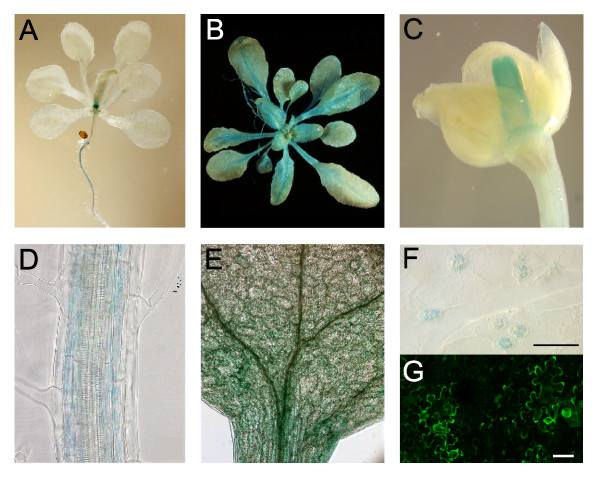
***CNGC20 *expression in roots and shoots**. GUS staining of plants carrying the *CNGC20p:GUS *construct was observed in roots of young seedlings (A), in petioles and tissue surrounding the veins of adult leaves (B), and carpels (C). On the cellular level, GUS staining was detected in the root cortex (D), mesophyll surrounding the veins (E), and in guard cells (F). (G) GFP fluorescence in epidermal cells after Agrobacterium-mediated transformation of *Nicotiana benthamiana *leaves with the *CNGC20p:GFP *construct. The bars represent 50 μm.

GUS staining in plants expressing the β-glucuronidase gene under control of the *CNGC20 *promoter was visible in the roots of young seedlings (Fig. 2A), but was more pronounced in shoot tissue of mature plants (Fig. 2B), as well as in the carpel (Fig. 2C) and crown leaves of flowers (not shown). While within the root, cortex tissue was stained, in the shoot *CNGC20 *gene expression was mainly observed in the mesophyll tissue surrounding the veins and in the petioles (Fig. 2B, E). Expression in guard cells could also be observed (Fig. 2F). Staining of epidermal cells, however, was weak. To test this, tobacco leaves were transiently transformed with a *CNGC20 *promoter-GFP fusion construct. Indeed, promoter activity was evident in epidermal cells (Fig. 2G).

Data in this study indicate specific expression patterns for both genes. Interestingly, CNGC19 is found in the vasculature, which is surrounded by CNGC20 expressing cells. Thus, the two genes may fulfil similar functions in different but adjacent tissues.

### *CNGC19 *and *CNGC20 *expression is regulated by salinity

**Figure 3:**
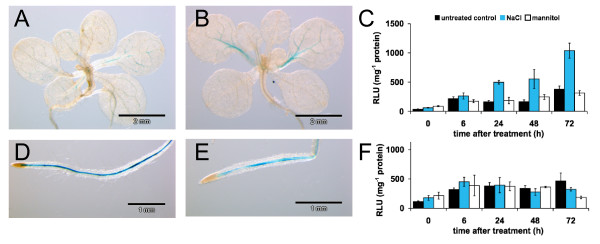
**Salt regulation of *CNGC19***. (A-E) GUS staining of 18-day old plants carrying the *CNGC19p:GUS *construct, which were grown in the absence (A, D) or presence (B, E) of 150 mM NaCl for 7 days. (C, F) Luciferase activity in *CNGC19p:LUC *transformed plants. Promoter activity was determined as relative luciferase luminescence units (RLU) in shoots (C) and roots (F) normalized to the respective protein content. Roots of 12-day old plants were either untreated (black bars), or treated with 200 mM NaCl (blue bars) or 300 mM mannitol (white bars). Luciferase activities were determined after stress application at time points indicated. Data represent mean ± SEM (n = 3).

Previous publications reported on the participation of nonselective cation channels in the plant's response to salt stress [17,18]. To investigate possible effects of salinity on *CNGC19 *and *CNGC20*, we monitored gene activities using reporter genes and quantitative RT-PCR. When plants transformed with *CNGC19p:GUS *were grown for one week on half-strength MS-agar in the absence or presence of 150 mM NaCl, GUS-staining of seedlings and whole plants revealed strong promoter activity in root tissue under both control and salt stress conditions (Fig. 3D, E). In the aerial parts of the plant, the staining intensity was increased in 65% (13 out of 20) of the plants tested after the salt stress period compared to controls (Fig. 3A, B).

Transgenic plants expressing the luciferase gene under the control of the *CNGC19 *promoter were used to determine the gene activity as the luciferase luminescence intensity normalized to the protein content of tissue extracts (relative luciferase luminescence). The relative luciferase luminescence in 12-day old plants was higher in root than in shoot tissue (Fig. 3C, F). The same approach was used to monitor gene regulation by NaCl treatment. After application of 200 mM NaCl to the root, *CNGC19 *gene activity increased only in the shoot (Fig. 3C) but not in the root (Fig. 3F). The increase continued during 72 hours of salt stress, corresponding to a steady upregulation of *CNGC19 *gene activity. In the presence of 300 mM mannitol, *CNGC19 *was not affected in the same manner, indicating that the response was specific and mainly due to the ionic rather than the osmotic component of the stress. These results are well in agreement with whole genome array data on cDNA isolated from 13-day old plants, which show a time-dependent accumulation of CNGC19 transcripts in the shoot [19]. In the root, transcript levels rose transiently within the first hour after salt treatment, but returned to control levels within 6 hours.

**Figure 4:**
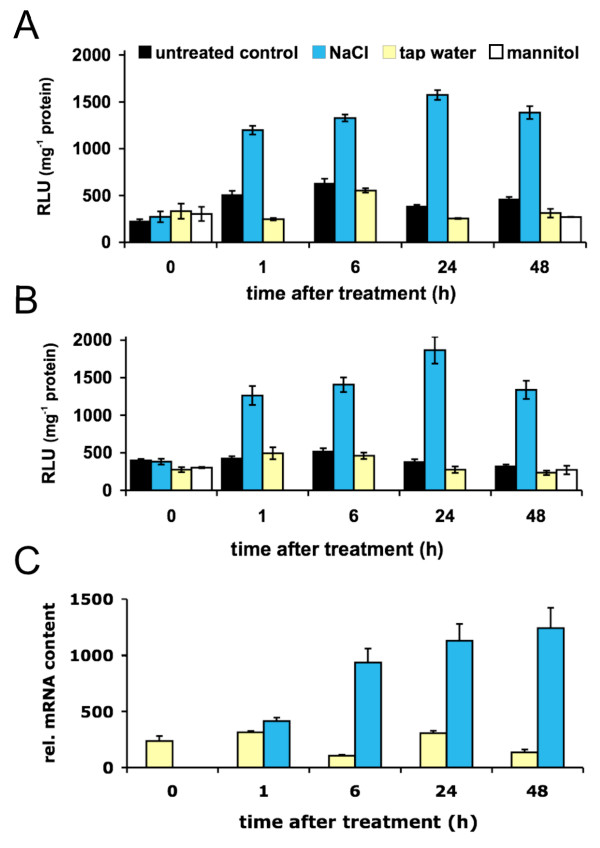
**Salt regulation of *CNGC20***. CNGC20 promoter activity was monitored in 6-week old plants, using the luciferase reporter (A, B) or quantitative RT-PCR (C). (A) Luciferase activity in leaves from plants carrying the *CNGC20p:LUC *construct, at indicated time points after treatment of the roots with 200 mM NaCl (blue bars), 300 mM mannitol (white bars), tap water (yellow bars) or in untreated material (black bars). Data represent mean ± SEM (n = 3). (B) Petioles of detached leaves were put into Hoagland Medium (black bars), 200 mM NaCl-, 300 mM mannitol-, or tap water-containing solution and luciferase activity was quantified at time points indicated. Data represent mean ± SEM (n = 3). (C) *CNGC20 *transcript levels in detached leaves after treatment with 200 mM NaCl or tap water. Transcript levels are expressed relative to the respective actin control. Data represent mean ± SEM (n = 7-8). Color code in (B) and (C) as in (A).

Kilian and colleagues [19] did not detect a salt-dependent regulation of *CNGC20*. We therefore decided to test the salt-sensitivity at developmental stages, where *CNGC20 *is predominantly expressed. 6-week old plants expressing luciferase under control of the *CNGC20 *promoter reported indeed a salt-dependent increase in *CNGC20 *expression (Fig. 4A, B). Application of 200 mM NaCl to roots led to a significant increase of luciferase in the leaves already within one hour, which then remained elevated during two days of constant salt stress (Fig. 4A). *CNGC20 *was not upregulated in control conditions or in the presence of 300 mM mannitol. Kinetics of the salt response of *CNGC20 *indicated that tissue not directly in contact with the stress responded rapidly. To test, whether this could be due to the uptake and translocation of salt to the shoot tissue, the stress was applied directly to the leaves by putting the petioles of detached leaves into the NaCl solution. As in the root treatment, *CNGC20 *in the shoot responded within one hour as indicated by the increase in relative luminescence, while control treatments had no effect (Fig. 4B). Salt-dependent regulation of *CNGC20 *was supported by quantitative RT-PCR experiments. Transcript abundance was assessed in detached leaves, revealing a salt-dependent increase of *CNGC20 *transcript in the shoot (Fig. 4C), which was significantly different from controls.

Together, promoter activities assessed by reporter genes and transcript levels determined by quantitative RT-PCR reported qualitatively similar results on the salt-dependent upregulation of *CNGC19 *and *CNGC20 *in leaves of mature plants. Our data point to a physiological response to the accumulation of NaCl itself rather than to an osmotic shock response. The results show that salt treatment of the root triggers a response of *CNGC20 *in the leaf. Such long-distance signaling from root to shoot might be mediated by hormones, such as abscisic acid (ABA) [20,21]. However, the response of isolated leaves demonstrated that salt perception and signal transduction can take place in the aerial parts of the plant. Thus, it appears likely that the regulation of *CNGC20 *depends on the direct transfer of NaCl to the shoot. It remains unclear in which cell types the signal perception takes place and whether this is the same for both genes. Since *CNGC19 *is expressed in phloem tissue and *CNGC20 *in mesophyll cells nearby the phloem, it is interesting to note that the *CNGC20 *induction kinetics saturates much earlier compared to that of *CNGC19*. Whether or not this is related to the time-dependent distribution of NaCl within the shoot remains to be clarified.

Salt stress, like many other abiotic stresses, can elicit a transient increase in cytosolic Ca^2+ ^[22]. In Arabidopsis seedlings, cGMP levels increased rapidly (*<*5 s) and to different degrees after salt and osmotic stress [23]. Interestingly, Donaldson and colleagues provided evidence that salt stress activates two cGMP signaling pathways - an osmotic, calcium-independent pathway and an ionic, calcium-dependent pathway. It is tempting to suggest that CNGC19 and CNGC20 might be suitable candidates taking part in these early responses, possibly linking cGMP- and Ca^2+^-signaling.

Increased expression of *CNGC20 *was detected quickly within one hour. *CNGC19 *responded a bit slower within 24 hours. The strong induction of the expression by NaCl implies a function in the adaptation to salinity. In this respect it is interesting that salt stress affects both genes mainly in the shoot, where most dramatic changes occur [1]. Control of Na^+ ^accumulation in the shoot is of major importance for the adaptation to salt stress. As most sensitive plants display poor ability to sequester Na^+ ^in leaf vacuoles, they have to rely on other mechanisms to cope with the Na^+ ^delivered to leaf cells. Both CNGC19 and CNGC20 represent possible Na^+ ^entry pathways into cells, and could participate in the Na^+ ^distribution within the leaf. For instance, CNGC19 could participate in Na^+ ^sequestration into phloem parenchyma cells and CNGC20 in Na^+ ^sequestration into the mesophyll of petioles. A translocation of Na^+ ^to petioles is known from species that tolerate salt [6,7], but might occur to a certain extent also in Arabidopsis. Since Na^+ ^is preferentially deposited in older leaves, a role in compartmentation is supported by the fact that *CNGC20 *is mainly expressed in older leaves.

Expression of *CNGC19 *was detected in the phloem, strengthening the hypothesis about a function in phloem loading and unloading. CNGC19 could be involved in Na^+ ^recirculation from shoots to roots, where Na^+ ^might be extruded, or at least in Na^+ ^redistribution between tissues. In the upper parts of the roots and in the stem, a direct transfer of sodium ions from xylem to phloem tissues is thought to play a role in the control of Na^+ ^translocation towards the shoot [24-27]. This would require Na^+ ^uptake into the phloem. Na^+ ^assays of the phloem sap revealed high concentrations up to 80 mM in some species [28], but the physiological significance of such data was interpreted contradictorily [29,30]. According to the expression pattern and expected ion channel characteristics, it is tempting to hypothesize that CNGC19, similar to AKT2/3 [31], might play a role in membrane potential stabilization and therewith might indirectly affect phloem (re)loading of metabolites.

### Phenotypical analysis of *cngc19 *and *cngc20 *mutants

**Figure 5:**
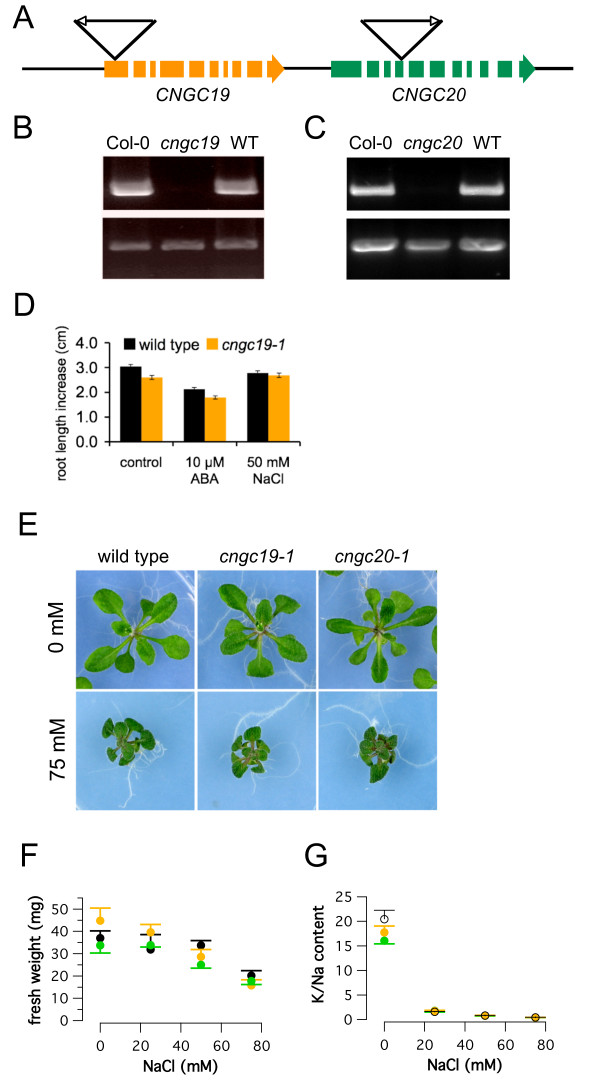
**Analysis of *cngc19 *and *cngc20 *T-DNA insertion lines**. (A) Genomic organization of *CNGC19 *and *CNGC20 *and the respective T-DNA insertions for *cngc19-1 *(SALK line 027306) and *cngc20-1 *(SALK line 129133). Exons are shown in bold. (B) Absence of *CNGC19 *mRNA from *cngc19-1 *plants (lane 2), but presence in Col-0 (lane 1) and a backcrossed *CNGC19 *wild type (WT, lane 3). *Upper traces*: PCR result using *CNGC19 *gene-specific primers, which amplified a 348 bp fragment downstream of the T-DNA insertion, *lower traces*: *Actin2 *primers were used as a control. (C) Corresponding PCR analysis of the T-DNA insertion line *cngc20-1 *with cDNA from Col-0 (lane 1), *cngc20-1 *(lane 2) and a backcrossed *CNGC20 *wild type (WT, lane 3). *CNGC20 *gene-specific primers, which span a 310 bp fragment downstream of the T-DNA insertion, were used. No PCR fragment was amplified from cDNA from homozygous *cngc20-1 *(lane 2). (D) Unchanged root growth of *cngc19-1 *plants. Root length increase of *cngc19-1 *and wild type seedlings were measured during a 7-day growth period, starting 4 days after stratification. Mutant (orange bars) and wild type (black bars) plants grew vertically on half-strength MS agar plates in the absence (control) or presence of 10 μM ABA or 50 mM NaCl. Data represent mean ± SEM (n = 30). (E) Absence of a salt-dependent growth phenotype in *cngc19-1 *and *cngc20-1*. Photographs show representative plants from wild type, *cngc19-1*, and *cngc20-1 *after a 12-day growth period in the absence or presence of 75 mM NaCl. (F) Fresh weight of wild type (black circles), *cngc19-1 *(orange circles), and *cngc20-1 *(green circles) shoots after a 12-day growth period in the presence of 0, 25, 50, or 75 mM NaCl. Data represent mean ± SEM (n = 6). The dry weight did also not differ significantly between wild type and mutants (not shown). (G) K/Na content in shoots of plants shown in (E) and (F) as a function of the applied salt-concentration. Data represent mean ± SEM (n = 6). Color code as in (F).

Arabidopsis T-DNA insertion lines from the Salk collection [32] were investigated with T-DNA insertions in the first (Salk_027306, *cngc19-1*) and fourth exon (Salk_129133, *cngc20-1*), respectively (Fig. 5A). PCR using cDNA isolated from wild type and mutants confirmed that *CNGC19 *and *CNGC20 *expression was virtually absent from the respective homozygous T-DNA mutants (Fig. 5B, C).

We analyzed the CNG channel mutants in conditions mimicking high salinity and in the presence of abscisic acid (ABA), which plays a crucial role in root-to-shoot and cellular signaling in response to salt stress [1]. Although *CNGC19 *is expressed in the root tissue of young seedlings, *cngc19-1 *displayed no root growth phenotype under control or saline (50 mM NaCl) conditions. The growth was also not affected by 10 μM ABA compared to the wild type (Fig. 5D). Similarly, the germination was indistinguishable from wild type in the presence of 50 mM NaCl or 10 μM ABA (data not shown). However, root growth is usually less affected than leaf growth during Na^+ ^toxicity, and the root elongation rate recovers remarkably well after exposure to NaCl or other osmotica [30]. Regarding the whole plant, it is primarily the mature leaf where Na^+ ^toxicity is manifested. As both *CNGC19 *and *CNGC20 *are upregulated in shoot tissue of salt-stressed plants, we compared the shoot growth of mutants and wild type. After a 5 day growth period on half-strength MS-agar, seedlings were transferred to agar plates containing 0, 25, 50, or 75 mM NaCl and grown for another twelve days. No salt-dependent phenotype could be observed for *cngc19-1 *and *cngc20-1 *mutants compared to the wild type (Fig. 5E, F).

To test if Na^+ ^or K^+ ^accumulation is affected in the mutants, contents were determined with ICP. Although the plants displayed a reduction of fresh weights with increasing NaCl content in the media (Fig. 5F), the K:Na content ratio of wild type and mutant shoots of plants grown for twelve days on plates containing 0, 25, 50 or 75 mM NaCl did not differ (Fig. 5G). These findings suggest that both uptake of sodium/potassium and extrusion of Na^+ ^are unaltered in the mutants.

Phenotypic characterization of loss-of-channel mutants does not allow deducing an explicit role of CNGC19 and CNGC20 during the nonselective uptake of Na^+ ^during salt stress. Due to their expression pattern, they could be involved in salt stress-dependent signal transduction or distribution of sodium throughout the plant. In the mesophyll and the root cortex, the specific role of CNGC20 might be masked by the activity of other nonselective cation channels having partially redundant functions. *CNGC20 *and *CNGC3*, for instance, are both expressed in the root cortex. *Cngc3 *loss-of-function mutants grew slightly better in the presence of 40 to 80 mM NaCl [13]. This together with short and long-term Na^+ ^influx experiments led to the conclusion that CNGC3 is functioning in sodium (and potassium) influx. Only during initial stages of salt stress, CNGC3 contributed considerably to Na^+ ^influx. Its participation in ion uptake in salinity-adapted plants seems to be limited [13].

In leaves, CNGC10 was located in the plasma membrane of mesophyll, palisade parenchyma and epidermal cells [14]. Mature plants of *CNGC10 *antisense lines were more sensitive towards salt stress and their shoots contained higher Na^+ ^concentrations compared with wild type [12]. GUS staining showed that *CNGC10 *expression in leaves is patchy; it comprised vascular tissue and mesophyll (B. Köhler, unpublished results). Thus, at least a partially redundant function to CNGC20 appears possible.

Apart from pollen-specific CNGCs, the CNGCs investigated so far show a broad expression pattern [13,14,16,33]. By comparison, expression of *CNGC19 *is relatively confined. Thus, further cell-type specific functional assays are required to assess its physiological role *in planta*.

## Conclusion

*CNGC19 *is expressed in the phloem and *CNGC20 *in the epidermis and the mesophyll, mainly in petioles. Upon salinity, both genes are upregulated within hours in the shoot, where most dramatic changes happen [1]. Salt-dependent regulation of *CNGC20 *occurred in the shoot, irrespective of whether NaCl was applied to the roots of intact plants or to the petioles of detached leaves. At first glance, it seems puzzling that a cell promotes the upregulation of genes encoding proteins that provide Na^+ ^entry pathways and therefore would contribute to increase the cytosolic sodium levels. However, under severe salt stress, CNG channels represent a fast and effective way to redistribute sodium throughout the whole plant. The fact that both *CNGC19 *and *CNGC20 *were upregulated by salt rather than by osmolytes indicates a role in salt adaptation. Therefore we propose a distributive role for CNGC19 and CNGC20 enabling the plant to cope with toxic effects caused by salt stress.

## Methods

### Plant material and growth conditions

*Arabidopsis thaliana *Col-0 ecotype and transgenic plants in Col-0 background were used. Plants were grown on soil in a growth chamber at a photoperiod of either 16 h (long day LD) or 8 h (short day SD). For sterile cultivation, seeds were sown on half-strength MS agar pH 5.8 containing 1% sucrose and 0.8% phytagar (Duchefa). For culture on sand (1-2 mm aquarium grit), nutrients were supplied by modified Hoagland medium [34], containing 1.25 mM KNO_3_, 1.5 mM Ca(NO_3_)_2_, 0.75 mM MgSO_4_, 0.5 mM KH_2_PO_4_, 50 μM KCl, 50 μM HBO_3_, 10 μM MnSO_4_, 2 μM ZnSO_4_, 1.5 μM CuSO_4_, 0.075 μM (NH_4_)_6_Mo_7_O_24_, 72 μM FeSO_4_, 89.28 μM EDTA, pH 6. In all conditions, plants grew at 22°C and about 80-100 μmol/m^2^sec light intensity.

### Generation of transgenic plants

For reporter gene studies, a 1.15-kb promoter region of the *CNGC20 *gene was introduced into the binary vector pVKH-35S-pA1 [35], where it replaced the 35S promoter in front of the *uidA *gene, resulting in the binary vector, pVKH-*CNGC20p:GUS*. Additionally, the promoter region was inserted into the destination vector pMDC206 [36], using gateway technology (Invitrogen). In case of *CNGC19*, a 1.82-kb promoter region was amplified by PCR using the primers PC19 for (5'-CCGCTCGAGAGCAACATGACAAACTTCTTC) and PC19rev (5'-CTAGCTAGCTTTTTATTTCAGAAACCCAAAATCTAGGGC). PCR fragments and the binary vector pGPTV-HPT [37] were cut with *Xho*I and *Nhe*I, and *Sal*I and *Xba*I, respectively, and ligated. The resulting plasmid was named pGPTV-*CNGC19p:GUS*. For luciferase studies, the luciferase^+ ^gene was introduced in pMDC206, where it replaced the GFP coding region, resulting in the new destination vector pMDC206-luc. A 1.26-kb promoter fragment of the *CNGC19 *gene and the *CNGC20 *promoter region were inserted into pMDC206-luc via gateway cloning. *Agrobacterium tumefaciens *strain GV3101 carrying the binary plasmids was used to transform *A. thaliana *Col-0 [38]. Transgenic plants were tested for reporter gene activities. Homozygous lines were produced for each construct.

### GUS histochemical assay

GUS staining followed the method of Jefferson et al. [39]. The plant tissue was cleared in 70% ethanol for 1-2 days. For vibratome sectioning, the tissue was embedded directly after staining into 5% agarose in PBS (0.8% NaCl, 0.02% KCl, 0.144% Na_2_HPO_4_, 0.024% KH_2_PO_4_, pH 7.4 HCl) and then cut into 30 μm-sections with a vibratome (Model 1500; The Vibratome Company, St. Louis, USA).

### Agrobacterium-mediated transient expression in *Nicotiana benthamiana*

Overnight cultures of *Agrobacterium tumefaciens *strain *C58C1 *transformed with the *CNGC20p:GFP *construct, and Agrobacterium strain *p19 *featuring a viral-encoded suppressor of gene-silencing were used for coinfiltration into the abaxial side of *Nicotiana benthamiana *leaves [40]. Confocal images were taken 4 days after infiltration using a Leica confocal microscope (TCS SP II; Leica Microsystems, Wetzlar, Germany). Fluorescence was excited with a UV argon laser at 488 nm, and emission of GFP was detected in the range from 497-547 nm. Emission of chlorophyll was collected at 644-731 nm and transmitted light was detected at 779-840 nm.

### Luciferase activity assay

Seedlings of a homozygous *CNGC19p:LUC *line were grown vertically on half-strength MS agar plates in LD conditions for 12 days. For stress application, 3 ml of solution containing either 200 mM NaCl, 300 mM mannitol, or tap water was applied to the root tissue. 50 mg samples of root and shoot tissue were harvested at different time points after treatment, homogenized and suspended in CCLR buffer (Promega Corp. Madison, USA). After 30 min incubation on ice, extracts were cleared by 30 min centrifugation at 4°C at 10.000 g and the supernatant was stored at -80°C.

*CNGC20p:LUC *transformed plants were grown for 6 weeks either on sand or on soil. Sand-cultured plants were salt-treated by replacing the modified Hoagland medium with medium supplemented with 200 mM NaCl or 300 mM mannitol. For direct stress treatment of the shoot, leaves of soil-grown plants were cut and petioles placed in 200 mM NaCl or 300 mM mannitol solution. Leaf extracts were prepared after the indicated incubation times.

The frozen luciferase extracts were thawed on ice and luciferase luminescence was determined in a 50 μl aliquot after addition of 150 μl LAR buffer (Promega), using an Orion II 96 microplate luminometer (Berthold Detection Systems GmbH, Pforzheim, Germany). Protein contents of the samples were determined using the Roti-Nanoquant kit (Carl Roth GmbH, Karlsruhe, Germany). After background subtraction, the relative luminescence (RLU) was determined by normalization to the total protein content.

### Quantitative RT-PCR

Total RNA of shoots of 9-week old plants was isolated using TRIZOL reagent [41]. First strand cDNA was prepared from 7.5 μg of RNA in a total volume of 10 μl using the RevertAid H Minus first-strand cDNA synthesis kit (Fermentas, St. Leon-Rot, Germany) and diluted for RT-PCR 20-fold in water. PCR was performed in a Rotogene 2000 (Corbett, Concorde, USA) with the LightCycler-FastStart Quanti Tect SYBR Green PCR Kit (Qiagen, Hilden, Germany), using the *CNGC20 *gene-specific primers (5'-CCTCGAACGCTCTTCTGTAAA and 5'-CTAGTTATAGCCTTTAGTTTGTA). Actin2 primers (5'-ATTTCAGATGCCCAGAAGTCTTGTT and 5'-GAAACATTTTCTGTGAACGATTCCT) were used to normalize the *CNGC20 *mRNA level to that of actin.

### Isolation of T-DNA insertion lines

Seeds of T-DNA insertion lines for *CNGC19 *(Salk_027306, named *cngc19-1*) and *CNGC20 *(Salk_129133, named *cngc20-1*) were obtained from the SALK institute (http://signal.salk.edu/cgi-bin/tdnaexpress, [32]). Homozygous mutants were genotypically identified through PCR using a gene-specific primer (CNGC19: 5'-TGCACATCCCTAATGTCCA; CNGC20: 5'-GATGGCCGATGACTAAAGC) in combination with a T-DNA border primer (5'-CTGGCGTAATAGCGAAGACG) and a PCR using a gene-specific primer pair (CNGC19: 5'-TGCCCTAGATTTTGGGTTTC and 5'-AAATACTCTTGTGTCAGCTGCTATG; CNGC20: 5'-TCCCCTCTTCTTCTTCCTCATAAA and 5'-AACCAGTAGGAGCTCTAACGTAAC). For determination of *CNGC19 *transcript levels, total RNA isolated from root tissue of 200 *Arabidopsis thaliana *plants vertically grown for 12 days on half-strength MS medium was transcribed into cDNA as described above. *CNGC19 *gene-specific primers binding downstream of the T-DNA insertion (5'-GAAACTTGGAACTTTGGAGC and 5'-CTACCAAACCAAACATCATCAT) were used to verify the lack of *CNGC19 *mRNA in *cngc19-1 *plants. *CNGC20 *transcript levels were assayed in total RNA isolated from leaves of 5-week old plants. PCR was carried out on transcribed cDNA with a *CNGC20 *gene-specific primer set binding downstream of the T-DNA insertion in *cngc20-1 *(5'-CCTCGAACGCTCTTCTGTAAA-3' and 5'-CTAGTTATAGCCTTTAGTTTGTA). Transcript levels in the mutants were compared to the ones in Col-0 wild type as well as in a backcrossed wild type line. Actin2-specific primers were used as controls in all reactions. No *CNGC19 *and *CNGC20 *transcripts were detected in *cngc19-1 *and *cngc20-1 *mutants, respectively.

### ICP Analysis

Shoot dry weights of wild type, *cngc19-1 *and *cngc20-1 *plants grown for 12 days in MS-agar containing 0, 25, 50 or 75 mM NaCl were determined after 72 h incubation at 60°C. K and Na content analysis was performed in an ICP emission spectrometer JY 70 Plus (Division d'Instruments S.A./Jobin, France) after solubilization of the plant material in 1 ml conc. HNO_3 _for 10 h at 170°C under pressure (10 bar) followed by a dilution step (1:10) in deionized water.

## Authors' contributions

PD, BK, and KP conceived the study and designed experiments. AK, BK, and PW performed the experiments and carried out analysis. PD, BK, AK, and KP wrote the manuscript. All authors read and approved the final manuscript.

## References

[B1] Munns R Tester M Mechanisms of salinity tolerance Annu Rev Plant Biol 2008 59 651 681 10.1146/annurev.arplant.59.032607.092911 18444910

[B2] Zhu JK Regulation of ion homeostasis under salt stress Curr Opin Plant Biol 2003 6 441 445 10.1016/S1369-5266(03)00085-2 12972044

[B3] Apse MP Aharon GS Snedden WA Blumwald E Salt tolerance conferred by overexpression of a vacuolar Na^+^/H^+ ^antiport in Arabidopsis Science 1999 285 1256 1258 10.1126/science.285.5431.1256 10455050

[B4] James RA Munns R von Caemmerer S Trejo C Miller C Condon TA Photosynthetic capacity is related to the cellular and subcellular partitioning of Na^+^, K^+ ^and Cl Plant Cell Environ 2006 29 2185 2197 10.1111/j.1365-3040.2006.01592.x 17081251

[B5] Karley AJ Leigh RA Sanders D Differential ion accumulation and ion fluxes in the mesophyll and epidermis of barley Plant Physiol 2000 122 835 844 10.1104/pp.122.3.835 10712547 PMC58919

[B6] Sibole JV Cabot C Poschenrieder C Barcelo J Ion allocation in two different salt-tolerant Mediterranean Medicago species J Plant Physiol 2003 160 1361 1365 10.1078/0176-1617-00811 14658389

[B7] Jeschke JPS Ionic interactions of petiole and lamina during the life of a leaf of castor bean (*Ricinus communis *L.) under moderately saline conditions J Exp Bot 1991 42 1105 1116 http://jxb.oxfordjournals.org/cgi/content/abstract/42/8/1051

[B8] Shi H Quintero FJ Pardo JM Zhu JK The putative plasma membrane Na^+^/H^+ ^antiporter SOS1 controls long-distance Na^+ ^transport in plants Plant Cell 2002 14 465 477 10.1105/tpc.010371 11884687 PMC152925

[B9] Olias R Eljakaoui Z Li J de Morales PA Marin-Manzano MC Pardo JM Belver A The plasma membrane Na^+^/H^+ ^antiporter SOS1 is essential for salt tolerance in tomato and affects the partitioning of Na^+ ^between plant organs Plant Cell Environ 2009 32 904 916 10.1111/j.1365-3040.2009.01971.x 19302170

[B10] Kaplan B Sherman T Fromm H Cyclic nucleotide-gated channels in plants FEBS Lett 2007 581 2237 2246 10.1016/j.febslet.2007.02.017 17321525

[B11] Mäser P Thomine S Schroeder JI Ward JM Hirschi K Sze H Talke IN Amtmann A Maathuis FJ Sanders D Phylogenetic relationships within cation transporter families of Arabidopsis Plant Physiol 2001 126 1646 1667 10.1104/pp.126.4.1646 11500563 PMC117164

[B12] Guo KM Babourina O Christopher DA Borsics T Rengel Z The cyclic nucleotide-gated channel, AtCNGC10, influences salt tolerance in Arabidopsis Physiol Plant 2008 134 499 507 10.1111/j.1399-3054.2008.01157.x 18823330

[B13] Gobert A Park G Amtmann A Sanders D Maathuis FJ Arabidopsis thaliana cyclic nucleotide gated channel 3 forms a non-selective ion transporter involved in germination and cation transport J Exp Bot 2006 57 791 800 10.1093/jxb/erj064 16449377

[B14] Christopher DA Borsics T Yuen CY Ullmer W Andeme-Ondzighi C Andres MA Kang BH Staehelin LA The cyclic nucleotide gated cation channel AtCNGC10 traffics from the ER via Golgi vesicles to the plasma membrane of Arabidopsis root and leaf cells BMC Plant Biol 2007 7 48 10.1186/1471-2229-7-48 17877833 PMC2031891

[B15] Borsics T Webb D Andeme-Ondzighi C Staehelin LA Christopher DA The cyclic nucleotide-gated calmodulin-binding channel AtCNGC10 localizes to the plasma membrane and influences numerous growth responses and starch accumulation in Arabidopsis thaliana Planta 2007 225 563 573 10.1007/s00425-006-0372-3 16944199

[B16] Frietsch S Wang YF Sladek C Poulsen LR Romanowsky SM Schroeder JI Harper JF A cyclic nucleotide-gated channel is essential for polarized tip growth of pollen Proc Natl Acad Sci USA 2007 104 14531 14536 10.1073/pnas.0701781104 17726111 PMC1964830

[B17] Maathuis FJ The role of monovalent cation transporters in plant responses to salinity J Exp Bot 2006 57 1137 1147 10.1093/jxb/erj001 16263900

[B18] Demidchik V Maathuis FJ Physiological roles of nonselective cation channels in plants: from salt stress to signalling and development New Phytol 2007 175 387 404 10.1111/j.1469-8137.2007.02128.x 17635215

[B19] Kilian J Whitehead D Horak J Wanke D Weinl S Batistic O D'Angelo C Bornberg-Bauer E Kudla J Harter K The AtGenExpress global stress expression data set: protocols, evaluation and model data analysis of UV-B light, drought and cold stress responses Plant J 2007 50 347 363 10.1111/j.1365-313X.2007.03052.x 17376166

[B20] Fricke W Akhiyarova G Veselov D Kudoyarova G Rapid and tissue-specific changes in ABA and in growth rate in response to salinity in barley leaves J Exp Bot 2004 55 1115 1123 10.1093/jxb/erh117 15047763

[B21] Fricke W Akhiyarova G Wei W Alexandersson E Miller A Kjellbom PO Richardson A Wojciechowski T Schreiber L Veselov D The short-term growth response to salt of the developing barley leaf J Exp Bot 2006 57 1079 1095 10.1093/jxb/erj095 16513814

[B22] Knight H Trewavas AJ Knight MR Calcium signalling in *Arabidopsis thaliana *responding to drought and salinity Plant J 1997 12 1067 1078 10.1046/j.1365-313X.1997.12051067.x 9418048

[B23] Donaldson L Ludidi N Knight MR Gehring C Denby K Salt and osmotic stress cause rapid increases in Arabidopsis thaliana cGMP levels FEBS Lett 2004 569 317 320 10.1016/j.febslet.2004.06.016 15225654

[B24] Davenport RJ Munoz-Mayor A Jha D Essah PA Rus A Tester M The Na^+ ^transporter AtHKT1;1 controls retrieval of Na^+ ^from the xylem in Arabidopsis Plant Cell Environ 2007 30 497 507 10.1111/j.1365-3040.2007.01637.x 17324235

[B25] Sunarpi Horie T Motoda J Kubo M Yang H Yoda K Horie R Chan WY Leung HY Hattori K Enhanced salt tolerance mediated by AtHKT1 transporter-induced Na unloading from xylem vessels to xylem parenchyma cells Plant J 2005 44 928 938 10.1111/j.1365-313X.2005.02595.x 16359386

[B26] Berthomieu P Conejero G Nublat A Brackenbury WJ Lambert C Savio C Uozumi N Oiki S Yamada K Cellier F Functional analysis of AtHKT1 in Arabidopsis shows that Na^+ ^recirculation by the phloem is crucial for salt tolerance Embo J 2003 22 2004 2014 10.1093/emboj/cdg207 12727868 PMC156079

[B27] Jeschke JPS Cation and chloride partitioning through xylem and phloem within the whole plant of *Ricinus communis *L. under conditions of salt stress J Exp Bot 1991 42 1105 1116 10.1093/jxb/42.9.1105

[B28] Jeschke WD Pate JS Temporal patterns of uptake, flow and utilization of nitrate, reduced nitrogen and carbon in a leaf of salt-treated castor bean (*Ricinus communis *L.) J Exp Bot 1992 43 393 402 10.1093/jxb/43.3.393

[B29] Munns R King RW Abscisic acid is not the only stomatal inhibitor in the transpiration stream of wheat plants Plant Physiol 1988 88 703 708 10.1104/pp.88.3.703 16666371 PMC1055648

[B30] Munns R Comparative physiology of salt and water stress Plant Cell Environ 2002 25 239 250 10.1046/j.0016-8025.2001.00808.x 11841667

[B31] Deeken R Geiger D Fromm J Koroleva O Ache P Langenfeld-Heyser R Sauer N May ST Hedrich R Loss of the AKT2/3 potassium channel affects sugar loading into the phloem of Arabidopsis Planta 2002 216 334 344 10.1007/s00425-002-0895-1 12447548

[B32] Alonso JM Stepanova AN Leisse TJ Kim CJ Chen H Shinn P Stevenson DK Zimmerman J Barajas P Cheuk R Genome-wide insertional mutagenesis of *Arabidopsis *thaliana Science 2003 301 653 657 10.1126/science.1086391 12893945

[B33] Köhler C Merkle T Roby D Neuhaus G Developmentally regulated expression of a cyclic nucleotide-gated ion channel from Arabidopsis indicates its involvement in programmed cell death Planta 2001 213 327 332 10.1007/s004250000510 11506354

[B34] Arnon DI Vitamin B1 in relation to the growth of green plants Science 1940 92 264 266 10.1126/science.92.2386.264 17740984

[B35] Baumann E Lewald J Saedler H Schulz B Wisman E Successful PCR-based reverse genetic screens using an *En-1*-mutagenised *Arabidopsis thaliana *population generated via single-seed descent Theor Appl Genet 1998 97 729 734 10.1007/s001220050949

[B36] Curtis MD Grossniklaus U A gateway cloning vector set for high-throughput functional analysis of genes in planta Plant Physiol 2003 133 462 469 10.1104/pp.103.027979 14555774 PMC523872

[B37] Becker D Kemper E Schell J Masterson R New plant binary vectors with selectable markers located proximal to the left T-DNA border Plant Mol Biol 1992 20 1195 1197 10.1007/BF00028908 1463855

[B38] Clough SJ Bent AF Floral dip: a simplified method for Agrobacterium-mediated transformation of *Arabidopsis thaliana* Plant J 1998 16 735 743 10.1046/j.1365-313x.1998.00343.x 10069079

[B39] Jefferson RA Kavanagh TA Bevan MW GUS fusions: beta-glucuronidase as a sensitive and versatile gene fusion marker in higher plants Embo J 1987 6 3901 3907 3327686 10.1002/j.1460-2075.1987.tb02730.x PMC553867

[B40] Voinnet O Rivas S Mestre P Baulcombe D An enhanced transient expression system in plants based on suppression of gene silencing by the p19 protein of tomato bushy stunt virus Plant J 2003 33 949 956 10.1046/j.1365-313X.2003.01676.x 12609035

[B41] Chomczynski P Sacchi N Single-step method of RNA isolation by acid guanidinium thiocyanate-phenol-chloroform extraction Analytical biochemistry 1987 162 156 159 10.1016/0003-2697(87)90021-2 2440339

